# Effect of Marine-Derived Ice-Binding Proteins on the Cryopreservation of Marine Microalgae

**DOI:** 10.3390/md15120372

**Published:** 2017-12-01

**Authors:** Hak Jun Kim, Bon-Won Koo, Doa Kim, Ye Seul Seo, Yoon Kwon Nam

**Affiliations:** 1 Department of Chemistry, Pukyong National University, Busan 48513, Korea; doakim@pknu.ac.kr (D.K.); sys4118@gmail.com (Y.S.S.); 2 Southeast Sea Fisheries Research Institute, National Fisheries Research and Development Institute, Namhae 52440, Korea; 89guti14@gmail.com; 3 Department of Marine Bio-Materials & Aquaculture, Pukyong National University, Busan 48513, Korea

**Keywords:** ice-binding proteins, ice recrystallization inhibition, cryoprotectant, slow-freezing, *Isochrysis galbana*, *Pavlova viridis*, *Chlamydomonas coccoides*

## Abstract

Ice-binding protein (IBPs) protect cells from cryo-injury during cryopreservation by inhibiting ice recrystallization (IR), which is a main cause of cell death. In the present study, we employed two IBPs, one, designated LeIBP from Arctic yeast, and the other, designated FfIBP from Antarctic sea ice bacterium, in the cryopreservation of three economically valuable marine microalgae, *Isochrysis galbana, Pavlova viridis*, and *Chlamydomonas coccoides*. Both of the IBPs showed IR inhibition in f/2 medium containing 10% DMSO, indicating that they retain their function in freezing media. Microalgal cells were frozen in 10% DMSO with or without IBP. Post-thaw viability exhibited that the supplementation of IBPs increased the viability of all cryopreserved cells. LeIBP was effective in *P. viridis* and *C. coccoides,* while FfIBP was in *I. galbana*. The cryopreservative effect was more drastic with *P. viridis* when 0.05 mg/mL LeIBP was used. These results clearly demonstrate that IBPs could improve the viability of cryopreserved microalgal cells.

## 1. Introduction

Ice-binding proteins (IBPs) are a class of protein that has an affinity to ice. Some IBPs, for example fish antifreeze proteins (AFPs) are a biological antifreeze, which bind to the ice surface and subsequently inhibit further growth of the ice crystal [1,2]. This behavior of IBP eventually lowers the freezing point of the solution, and hence creates a gap between freezing and melting points [3]. This activity is called thermal hysteresis (TH). TH is physiologically meaningful to organisms such as fish and insects, whose IBP’s main function is to prevent the organisms from freezing. The other important function of IBPs is ice recrystallization inhibition (IRI) [4,5,6,7,8,9,10]. Even below subfreezing temperatures, psychrophilic organisms inhabiting cold environments, such as alpine, Arctic, and Antarctica, experience relatively wide temperature fluctuations. These temperature fluctuations cause smaller ice grains to combine one another to form larger ones, which is thermodynamically favorable [11,12]. This process is called ice recrystallization (IR). IR phenomenon may be detrimental to organisms due to the freezing injury in the cell membranes, and dehydration [4,5,6,7,8,9,10]. IBPs situated in the interface between the ice grain boundaries bind to the ice grain surface, inhibit its growth [7], and increase the ice porosity in sea ice by changing the ice microstructure [8]. Therefore, IRI of IBPs seems to be essential for the cold-tolerant organisms [4,5,6,7,8,9,10]. IRI activity of IBPs, usually expressed as the endpoint concentration of its activity, is varied. Some IBPs can inhibit this process at very low concentration, which is beneficial to the organisms that are inhabiting those environments [4,5,7,13,14,15]. IRI of IBPs has enabled these biomaterials to be developed for a potential biological cryoprotective agent or cryoprotectant (CPA) (see references in [1]).

Recently, we have identified two IBPs: one (designated as LeIBP) from the Arctic yeast *Glaciozyma* sp. [16,17] and the other (designated as FfIBP) from the Antarctic sea ice bacterium, *Flavobacterium frigoris* [18,19]. Two IBPs share 56% sequence identity and have almost the same β-helical structure. They belong to the same β-helical family of IBPs. Since both of the IBPs are produced by organisms overwintering in a brine channel and have N-terminal signal peptide, their main role seems to protect the organisms themselves from freezing damage [16,20]. Both of the IBPs are known to possess TH and IRI activities. Despite their high similarity in primary and tertiary structure, LeIBP is moderately active (0.34 °C at 10.8 mg/mL) in TH [17], while FfIBP is hyperactive (2.2 °C at 0.13 mg/mL) [18]. This is because of the fact that FfIBP has more prominent ice-binding regular motifs (T-A/G-X-T/N motif) and ice-binding residues arrayed regularly on its ice-binding site than LeIBP [18]. However, their IRI showed opposite results [14,16]. LeIBP exhibited IRI down to 0.001 mg/mL concentration (37 nM), while FfIBP did to 0.028 mg/mL (2.5 μM). This indicates that TH activity is not necessarily proportional to IRI [19,20,21,22]. IRI activity of LeIBP is relatively high compared to fish and other β-helical IBPs [1]. Recently, a few bacterial IBPs having almost identical β-helical structure to LeIBP and FfIBP were reported [10,23]. Mangiagalli et al. demonstrated that a β-helical IBP (EfcIBP) from bacterial symbionts of the ciliate *Euplotes focardii* has IRI activity at 2.5 nM [10]. Even though the IRI activity analysis slightly differed between two IBP cases, the IRI activity of EfcIBP appears to be higher than LeIBP. Muñoz et al. also showed three IBPs identified from Antarctic microorganism can protect cellular structures of frozen food [23]. A large body of evidence demonstrated that IBPs with higher IRI activity could improve the cryopreservation efficiency of various biological samples [1]. The IRI activity of LeIBP has been utilized to improve the cryopreservation efficiency of red blood cells [15], mammalian cells [24], oocytes and ovarian tissues [25,26], diatoms [27], and sperm [28]. These attempts have met with some success. However, with an exception [25], FfIBP was less effective than LeIBP in cryopreservation.

Microalgae have great biotechnological potential for pharmaceutics, cosmetics, biomass, biofuel, as well as aquaculture diet [29,30,31,32]. In this study, we attempted to cryopreserve three mesophilic marine microalgae that were using both IBPs *Isochrysis galbana*, *Pavlova viridis*, and *Chlamydomonas coccoides*. Of the three marine microalgae used in the present study, two species, *I. galbana* and *P. viridis* belong to Haptophyceae, and *C. coccoides* to Chlorophyceae. We chose these species not only in that they are economically very valuable species as a fundamental live feed in fish and shellfish hatcheries [33,34,35,36,37,38], but also that they may be appropriate algal candidates to manifest the IRI activity of IBPs, since *I. galbana* and *P. viridis* lack distinct cell wall [39,40,41,42], while *C. coccodies* has a cell wall that is composed of microfibrillar layer of cellulose and glycoproteins [43]. As the aquaculture industry has grown globally [44], in the hatcheries the demand for culturing and maintaining marine microalgae, including the three species that were used in this study as food for juveniles has continuously increased [45]. Therefore, preserving these species as culture starters or biomass reserves has been desired. Like most microalgae, these species have been maintained by the most labor-intensive serial sub-culturing in many laboratories and hatcheries worldwide. The serial maintenance is not only costly, but is also likely to cause genetic drift, contamination, and the loss of their nutritional value as aquaculture feed [29,46,47]. Hence, cryopreservation of many microalgae has been attempted [29,45,46,47,48,49,50,51,52,53,54,55], but only fractions of numerous microalgal species have been successfully cryopreserved. In addition, the cryopreservation protocol varies from species to species [27,45,46,47,48,49,50,53,54,55]. Establishing a microalgae specific protocol is worth attempting.

In this study, we evaluated the cryoprotective effect of two IBPs, LeIBP (Accession no. GQ336995) and FfIBP (Accesion no. JQ712389), on commercially important marine microalgae during cryopreservation. Briefly, a CPA was selected, based on its toxicity, and was used to prepare freezing media containing IBPs, IRI of IBPs in the freezing media was evaluated, and the viability of microalgae was assessed and discussed.

## 2. Results and Discussion

### 2.1. Effect of CPAs on Unfrozen Marine Microalgae

**Figure 1:**
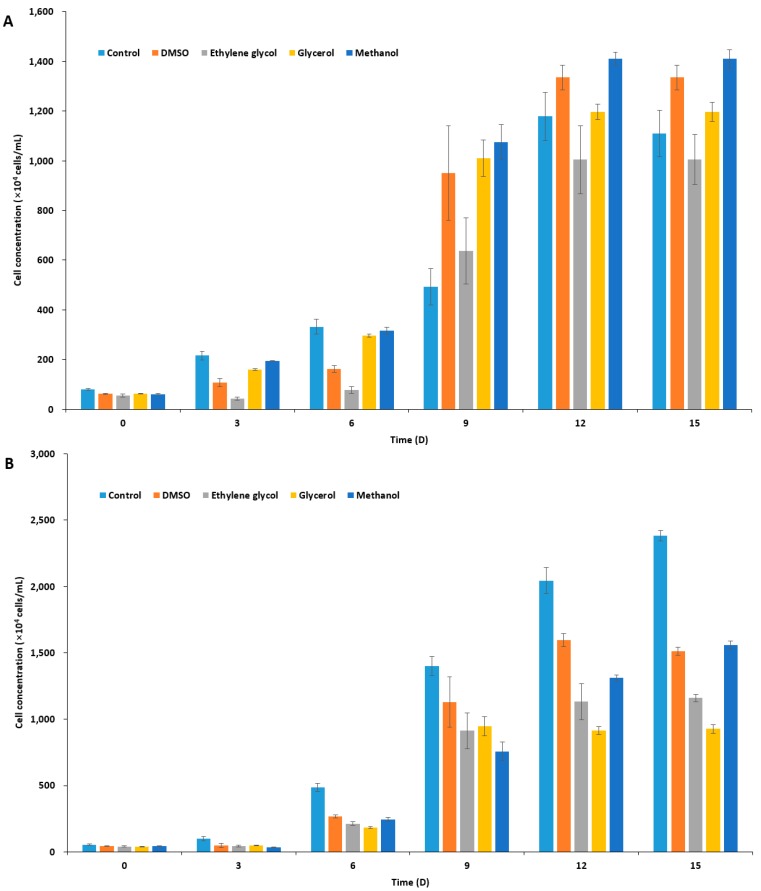

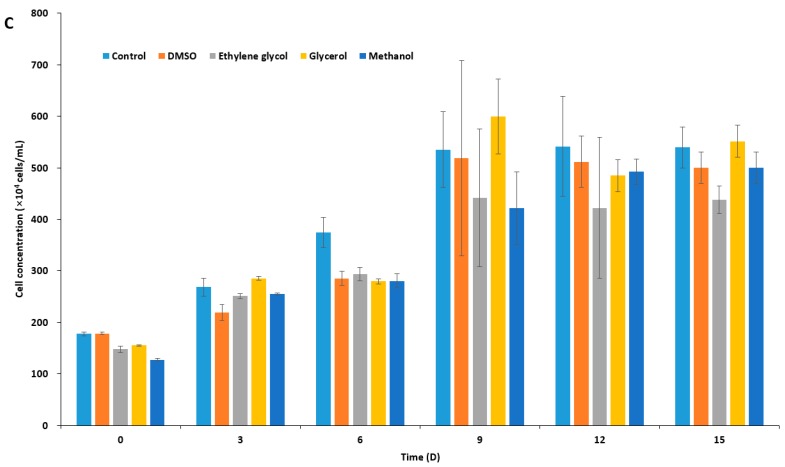
Concentration of cryoprotectant (CPA)-treated microalgal cells over time. After CPA treatment (10% each) for 10 min, cells were washed three times with sea water, inoculated to fresh f/2 media, and were cultivated for 15 days. (**A**) *Isochrysis galbana*; (**B**) *Pavlova viridis*; and (**C**) *Chlamydomonas coccoides*.

In order to select an appropriate CPA for *I. galbana, P. viridis,* and *C. coccoides*, based on the toxicity of the agent on the species, we treated microalgal cells with 10% four different CPAs in f/2 medium (Supplementary Table S1): dimethyl sulfoxide, DMSO; ethylene glycol, EG; glycerol, Gly; and, methanol, MeOH. After 10 min incubation and washing, the residual concentration of CPAs in the culture should be by far less than 0.1%. Measurements of microalgal cell concentration indicated two haptophyte species to *I. galbana* and *P. viridis* showed similar tolerance to all four of the CPAs, being most tolerable to DMSO and MeOH, while *C. coccoides* was tolerable to three CPAs, but least tolerable to EG (Figure 1). However, *P. viridis* was most susceptible to CPA treatment when compared to the other two species, illustrating a significant decrease in cell concentration as compared to untreated control cultures (Figure 1B). In the cryopreservation of microalgae, the choice of CPA varies from species to species, and some CPAs gave conflicting results with the same species under slight different cryopreservation protocol [27,29,45,46,48,50,53,54,55,56]. In the present study, we chose DMSO as a penetrating CPA, because not only DMSO has been most commonly used, but all of the three marine species seem to be adapted to the presence of nano to micromolar range DMSO in marine environment [57,58]. These marine species produce dimethylsulfoniopropionate, dimethylsulfide, and probably DMSO, which plays pivotal role in the sulfur cycle and function as an osmolyte [58], an anti-oxidant [59], and a CPA [60].

### 2.2. Ice Recrystallization Inhibition of Marine IBPs in Freezing Media

**Figure 2:**
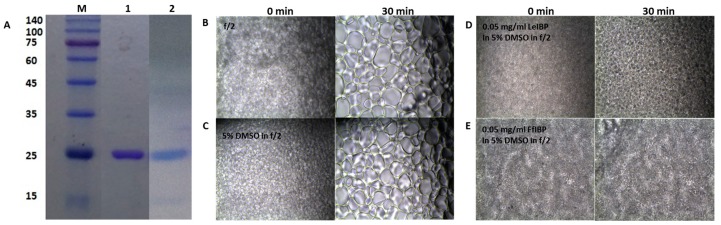
(**A**) Sodium dodeyl sulfate polyacrylamide gel electrophoresis (SDS-PAGE) (12%) analysis of purified recombinant IBPs and (**B**–**E**) ice recrystallization inhibition of ice-binding proteins (IBPs) in freezing media. (**A**) SDS-PAGE of purified recombinant IBPs. M: molecular weight makers; Lane 1, purified LeIBP; and Lane 2, purified FfIBP. The molecular weights of markers are listed in kDa to the left. (**B**–**E**) Ten μL of solution was dropped onto a prechilled aluminum block and a thin ice disc was formed. Subsequently, the ice disc was annealed for 30 min on the cold stage held at −6 °C. During the annealing, ice recrystallization occurs. The images captured at 0 (left column) and 30 min (right column) were presented. (**B**) f/2 alone, (**C**) 5% DMSO in f/2, (**D**) 0.05 mg/ mL LeIBP, and (**E**) 0.05 mg/mL FfIBP in 5% DMSO/f/2 freezing media.

The freezing media for the marine microalgae was composed of 10% DMSO in f/2 media in the absence or presence of the IBPs. IR is known as a major cause of cell death during cryopreservation [61]. Recombinant IBPs that were used in this study were shown in Figure 2A. The supplementation of IBPs in the freezing media was expected to add the IRI activity to it. The IRI assay showed that f/2 medium itself has no IRI activity (Figure 2B), when compared to the other freezing media. The grain sizes of its ice grew larger as time lapsed. DMSO in f/2 alone inhibited the growth of ice grain to some extent (Figure 2C). As the concentration of DMSO increased, the mean grain size slightly decreased, indicating that the IRI is concentration-dependent to certain degree (data not shown). This result corroborates the reports by the Ben group [62,63,64] and the Kim group [24]. Previously, we demonstrated that LeIBP held the IRI activity in the presence of DMSO [24]. We further examined the IRI of LeIBP and FfIBP in f/2 media containing 10% DMSO. The final concentration of IBPs in the freezing media was 0.05 and 0.1 mg/mL, which were well above their IRI endpoint, a concentration below which no IRI is observed (0.001 and 0.069 mg/mL for LeIBP and FfIBP, respectively). We chose these concentrations based on the previous reports that LeIBP was effective at 0.1 mg/mL in 10% DMSO in cryopreservation [24,25,27]. As expected, both of the IBPs in the freezing media showed IRI activity that was indistinguishable to those that were exhibited in the other buffers (Figure 2D,E). This implies that both of the IBPs are highly likely to remain active as an IR inhibitor in freezing media.

### 2.3. Viability of Cryopreserved Microalgae

**Figure 3:**
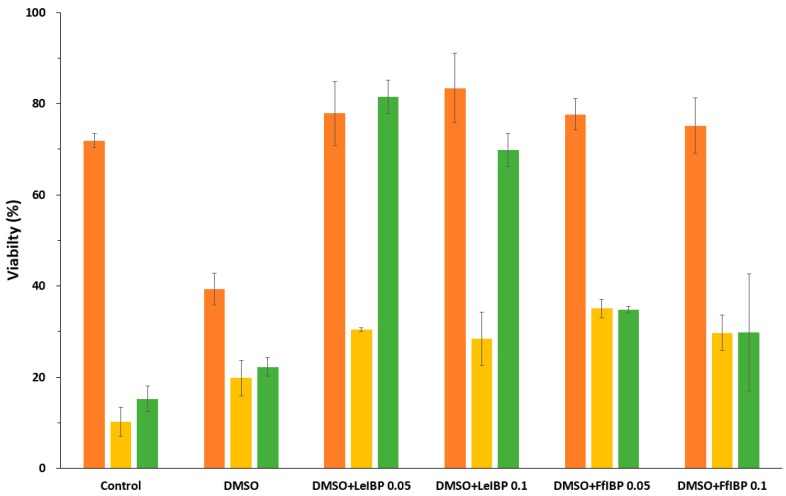
Post-thaw (0 h) viability of cryopreserved microalgal cells. Immediately after thawing, cells were washed three times and viable cells were counted as described in Materials and Methods. Results are expressed as % of cryopreserved cell density. *Isochrysis galbana*, was displayed in orange, *Pavlova viridis* in yellow, and *Chlamydomonas coccoides* in green.

Microalgal cells were cryopreserved in the absence and presence of IBPs using a two-step slow freezing protocol. As shown in Figure 3, the supplementation of IBPs significantly increased the post-thaw viability immediately after thawing in all of the cases when compared to untreated control and 10% DMSO alone. The effect of IBP was drastic in *I. galbana* and *C. coccoides,* but was moderate in *P. viridis*. Arctic yeast-derived LeIBP was more effective in *P. viridis* and *C. coccoides*, while Antarctic bacterium-originated FfIBP seemed more effective in *I. galbana*. Two concentrations showed no significant difference in viability, except for the *P. viridis* case. However, a lower concentration (0.05 mg/mL) was slightly effective in most cases except for FfIBP in *I. galbana*. This result somehow coincides with a number of observations that higher concentrations of IBP decreased the viability of cryopreserved cells [15,24,25,65,66,67].

**Figure 4:**
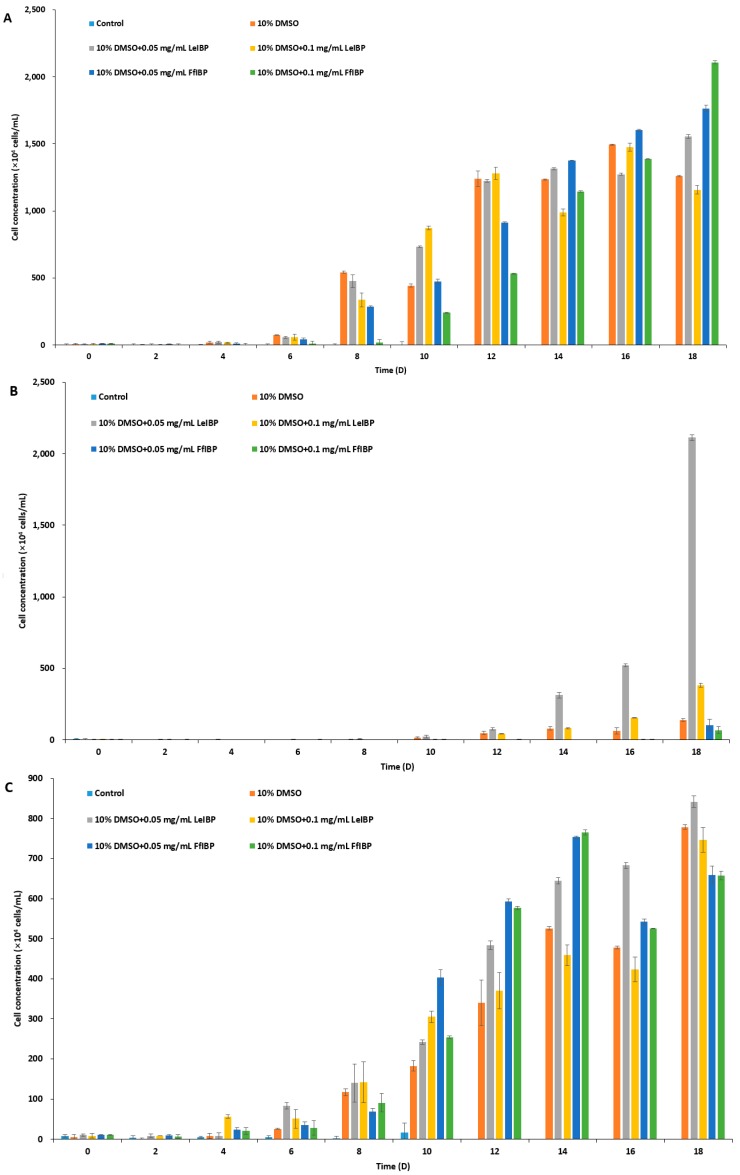
Growth of post-thawed *Isochrysis galbana* (**A**); *Pavlova viridis* (**B**); and *Chlamydomonas coccoides* (**C**). Thawed cells were inoculated in fresh media and culture densities were measured every other day. Each data point represent the mean ± SD of triplicate cultures.

The results of experiments with cell growth after thawing revealed that *I. galbana* and *C. coccoides* were successfully cryopreserved in 10% DMSO with and without IBPs (Figure 4A,C), while *P. viridis* was only cryopreserved successfully in the presence of LeIBP (Figure 4B). As shown in Figure 4A, *I. galbana* cryopreserved without CPA did not survive the freeze-thaw process. *I. galbana* cryopreserved in 10% DMSO did not showed almost any growth for the first six days, but its growth was recovered on the 8th day, and reached cell concentration as high as those that were cryopreserved in the presence of IBPs. The cell concentration of *I. galbana* treated with LeIBP increased faster than those treated with FfIBP for the first 12 days, but almost reached stationary phase till the end of the experiment. A lower concentration (0.05 mg/mL) of LeIBP was more beneficial. FfIBP-treated algal cells reached exponential growth slower than LeIBP-treated cells, but reached the highest cell density in 0.1 mg/mL FfIBP samples. In the case of Chlorophyta *C. coccoides* (Figure 4C) the supplementation of IBPs showed no beneficial effect when compared to DMSO alone. The IBPs seemed to affect growth in the lag phase, but eventually displayed no significant difference. The cell concentration of *C. coccoides* cryopreserved in 10% DMSO alone at the end of the experiment was almost similar to those that were treated with LeIBP, and slightly higher than those that were treated with FfIBP. Treatment of LeIBP was slightly more effective than that of FfIBP. The cell concentration was in order from high to low: 0.05 mg/mL LeIBP, 0.1 mg/mL LeIBP, 0.05 mg/mL FfIBP, 0.1 mg/mL FfIBP. Unlike the other two species, *P. viridis* was apparently hard to cryopreserve using the present protocol without the aid of LeIBP (Figure 4B). *P. viridis* cryopreserved without CPA, with 10% DMSO, and with 10% DMSO/FfIBP almost did not survive the freeze-thaw process: those samples had a long lag phase and very low growth rates. By contrast, *P. viridis* that was cryopreserved in the presence of LeIBP showed a significant post-thaw growth, even though its growth was delayed when compared to the other two species cases. *P. viridis* treated with 0.05 mg/mL LeIBP reached a higher cell concentration than that with 0.1 mg/mL LeIBP. Cell concentration remained very low for the first 12 days, but increased from the 14th day till the end of experiment. As illustrated with CPA-untreated control in three marine microalgae, the freezing and thawing process causes cryo-damage to cells. It is known that IR is one of the main causes of cell damage associated with cryopreservation [61]. IBPs present extracellularly in freezing media can effectively suppress restructuring of “extracellular” ice during the thawing process, eventually protecting cells from damage [1]. Some IBPs can inhibit IR significantly even at a very low concentration [2,68,69], which is not comparable with other nonpenetrating extracellular CPAs, such as PVP, HES, and dextran [70,71]. In all three microalgae cryopreservation, our data revealed that the supplementation of IBP can improve cell viability to some extent when compared to 10% DMSO alone, which reflects that IBPs inhibit the extracellular IR process during thawing. Rhodes et al. [46] successfully cryopreserved *I galbana*, *P. viridis*, and *C. coccoides* with either 10 or 15% DMSO with a similar freezing method to this study. Unfortunately, however, we could not compare our result with theirs since they provided no viability or growth curve in detail. In the present study, the cryopreservative effect of IBPs on microalgae was more prominent in two haptophyta than chlorophyta. This can be explained by the cell structure. Three marine microalgaes that were used in this study are of similar cell size but with different cell wall or scale structures. The chlorophyta has a well-defined cell wall [43], but two haptophyta species have no cell wall. The haptophyta have cell coverings called scales: dense layers of body scales for *I. galbana* [37,42] and small and relatively sparse knob shaped scales for *P. viridis* [42]. The haptophyta, *I. galbana* and *P. viridis,* are more fragile than *C. coccoides* [38,42], and *P. viridis* is most fragile of two haptophyta [37,38,42]. It can be speculated that the fragility makes them more vulnerable to the IR phenomenon. Therefore the cryoprotective effect is more drastic in *P. viridis*.

Of the two IBPs used in the present study, LeIBP seems to be more effective in cryopreservation. Especially at 0.05 mg/mL, LeIBP showed a better cryopreservative effect than the other experimental conditions. This result is in accord with other reports using the same IBPs [15,24,25,26,27]. As mentioned above, these IBPs belong to the same type, however, the cryopreservative effect of LeIBP was superior to that of FfIBP [25,26]. The reasons for better performance of LeIBP when compared to other IBPs remains to be elucidated.

## 3. Materials and Methods

### 3.1. Chemicals and Ice-Binding Proteins

All of the chemicals that were used in this study were purchased from Sigma Chemical Co. (St. Louis, MO, USA) The ice-binding proteins were prepared, as described elsewhere [18,72]. Briefly, the LeIBP originated from *Glaciozyma* sp. was recombinantly expressed in the methylotrophic yeast *Pichia pastoris* X33 cells containing pPICZαA harboring the mature LeIBP gene. The yeast cells were grown at 25 °C for two days in a flask containing yeast-peptone-dextrose medium. The LeIBP was induced by adding 5 mL of methanol daily for 6 days. After centrifugation at 8000× *g* for 10 min, the supernatant was loaded on to the Q-Sepharose FF column (GE Healthcare, Little Chalfont, UK) and eluted with 50 mM Tris-HCl buffer (pH 8.0) with salt gradient of 0 to 1 M NaCl. The elute fractions were pooled, concentrated, and loaded onto a Superdex 200 size-exclusion column (GE Healthcare) equilibrated with 50 mM Tris-HCl, pH 8.0, and 150 mM NaCl at a flow rate of 1 mL/min. The recombinant FfIBP was produced in the *Escherichia coli* strain BL21 (Invitrogen, Carlsbad, CA, USA) expression system. The *E. coli* strain harboring the FfIBP expression vector was grown at 37 °C in LB medium containing 100 mg/L ampicillin. When OD_600_ of the culture reached 0.6, the final concentration of 1 mM IPTG was added into the culture. The culture was then shifted to and maintained at 16 °C for another 18 h to induce the FfIBP. The cells were harvested by centrifugation 8000× *g* for 15 min. The bacterial pellet was sonicated in 50 mM Na_2_HPO_4_, pH 8.5, 300 mM NaCl, and 5 mM imidazole. The lysated was loaded onto a Ni-NTA agarose column pre-equilibrated with lysis buffer. The FfIBP was eluted from the column with a buffer containing 400 mM imidazole. After the cleavage of the 6X His tag using factor Xa, finally FfIBP was purified using the Superdex 200 size-exclusion column. The purified IBPs were confirmed by sodium dodecyl sulfate-polyacrylamide gel electrophoresis. The protein concentrations were determined from OD_280_ measurements with the calculated extinction coefficients of 26,930 M^−1^·cm^−1^ and 22,585 M^−1^·cm^−1^ for LeIBP and FfIBP, respectively.

### 3.2. Microalgae Strains and Culture Conditions

Two marine haptophyte, *Isochrysis galbana,* and *Pavlova viridis* were obtained from Southeast National Fisheries Institute, Namhae, Korea and one green algae *C. coccoides* Butcher (KMMCC-1755) from Korea Marine Micoalgae Culture Collection. Cells were maintained and cultured in flasks in f/2 medium at 20 °C with 20–30 μmol photons·m^−2^·s^−1^ in a 10:14 light/dark (L/D) photoperiod. Subculture was performed once per month when the cell density reached 10^6^ cells/mL.

### 3.3. Ice Recrystallization Inhibition (IRI) Assay

IRI was assessed using a splat cooling assay as described by Knight el al. [73]. All solutions were prepared in f/2 medium. Briefly, 10 μL of solution containing different amounts of IBPs in 2.5% and 5% DMSO solutions was dropped onto a polished aluminum plate that was pre-chilled by dry ice from a height of 2 m. As the droplet splatted onto the aluminum plate, it flash froze as an ice disc of approximately 1 cm in diameter and 20 μm in thickness. The disc was placed between two coverslips and then transferred to a Linkam LTS120 cold stage (Linkam Scientific Instruments Ltd., Surrey, UK) held at −6 °C. The ice disc was annealed for 30 min and its images were captured at 0 and 30 min by the Linkam Imaging Station. The f/2 medium was used as a control. To evaluate IRI activity quantitatively, images of the ten largest ice crystals was obtained and the mean grain size (MGS) of them was further analyzed by image J software (NIH) [74]. An inverse correlation between IBP concentration and IRI was plotted.

### 3.4. Toxicity of Four Penetrating CPAs

The toxicity of different cryoprotectants (CPAs) to the microalgae strains was evaluated. Ten % of four CPAs (dimethyl sulfoxide, DMSO; ethylene glycol, EG; glycerol, Gly; methanol, MeOH) was prepared in f/2 medium. Marine microalgae were collected using centrifugation at 3000× *g* for 10 min at 4 °C. The cells were resuspended in one ml of each of the four CPAs solutions at a concentration of 1 × 10^6^ cells/mL, incubated 10 min at room temperature, washed with autoclaved seawater three times, and then collected by centrifugation. The cell pellet was suspended in 50 mL of f/2 medium and cultured for 15 days as described above. Cells were counted every two days using a Thoma hemocytometer under a light microscope. Each measurement was done in triplicate.

### 3.5. Cryopreservation and Viability of Marine Microalgae

All of the microalgal cells in the exponential growth phase were harvested by centrifugation at 1200× *g* for 5 min at 12 °C. Harvested microalgal cells were resuspended at a concentration of 1 × 10^6^ cells/mL in five different freezing media: (1) 10% DMSO, (2) 10% DMSO and 0.05 mg/mL LeIBP, (3) 10% DMSO and 0.1 mg/mL LeIBP, (4) 10% DMSO and 0.05 mg/mL FfIBP, and (5) 10% DMSO and 0.05 mg/mL FfIBP. All freezing media were prepared in f/2 media. Microalgae was cryopreserved by two-step freezing method in a controlled rate freezer (Kryo 560, Planner, Middlesex, UK) and stored in the liquid nitrogen tank (VHC 35, Tailor Wharton, NJ, USA). Briefly, 1.8 mL cryogenic vials (Cryotube Nunc, Denmark) containing one mL (1 × 10^6^ cells/mL) of cells were placed in the controlled rate freezer. The vials were then cooled from 20 to 0 °C at a rate of −5 °C/min and further cooled to −40 °C at a rate of −1 °C/min. The vials were held at to −40 °C for 10 min before being transferred into liquid nitrogen (−196 °C). The vials were rapidly plunged to the liquid nitrogen tank and stored for 30 days. Thawing was carried out by immersing the cryogenic vials in a 30 °C water bath for 2 min 30 s with intermittent shaking. Then, the vials were centrifuged at 1200× *g* at 12 °C for 5 min. After decanting the supernatant containing the freezing media the cells were washed three times with autoclaved seawater to remove CPAs. The microalgal cells were resuspended in 35 mL of fresh f/2 medium and cultured for 18 days, as described above. Viability assays were conducted every other day. The samples were stained with Evans blue to evaluate the viability. Cultured microalgal cells were mixed with 0.1% (*w*/*v*) Evans blue dye in a 1:1 ratio, and incubated for 10 min at 12 °C in the dark. Viable cells were counted using a Thoma hemocytometer under a light microscope. As a positive dead control, cultured cells were boiled and stained with Evans blue. Each measurement was done in triplicate.

### 3.6. Statistical Analysis

Student’s *t*-test was conducted using Excel software (Microsoft, Redmond, WA, USA) to determine the significant differences, accepting *p* < 0.01 as significant. All of the experiments were carried out independently and repeated in triplicate; data were expressed as mean ± 1 SD.

## 4. Conclusions

In conclusion, IBPs, which are naturally occurring strong IR inhibitors, are proven to improve the viability of microalgal cells. IR is a main cause of cryo-injury during cryopreservation. IRI of IBPs remained active in the freezing media containing 10% DMSO in f/2. The cryopreservative effect of two IBPs on algal cells varied from species to species. The presence of IBP in freezing media increased the post-thaw viability and growth drastically in *P. viridis*, and, moderately in *I. galbana*; however, it was more or less ineffective in *C. coccoides*. In most cases, the lower amount (0.05 mg/mL) IBPs were usually more effective than the higher amount (0.1 mg/mL) IBPs. To our best knowledge, this is the first report of marine IBPs being used for the cryopreservation of marine microalgae other than diatom species. Taken together, these observations provide that IBPs can be used as a potent CPA or CPA supplement for many different types of cells.

## Supplementary Materials

The following is available online at www.mdpi.com/1660-3397/15/12/372/s1, Table S1: Composition of Guillard f/2 medium.
