# Supplementary material for: Effect of Marine-Derived Ice-Binding Proteins on the Cryopreservation of Marine Microalgae
Source: Mar Drugs. 2017 Dec 1;15(12):372. doi: 10.3390/md15120372 (PMC5742832; doi:10.3390/md15120372)
Supplement: Supplementary file 1 [file marinedrugs-15-00372-s001.pdf]

Supplementary data for

Effect of marine-derived ice-binding proteins on the cryopreservation of marine microalgae

Hak Jun Kim [1,] *, Bon-Won Koo [2], Doa Kim [1], Ye Seul Seo [1], and Yoon Kwon Nam [3]

Supplementary Table S1. Composition of Guillard f/2 medium [1]

Compound	Stock Solution (per liter)	Quantity per liter	Molar Concentration in Final Medium
$NaNO_3$	75 g	1 ml	8.83×10^{-4} M
$NaH_2PO_4.2H_2O$	5 g	1 ml	3.63×10^{-5} M
$Na_2SiO_3.9H_2O$	30 g	1 ml	1.06×10^{-4} M
Trace elements		1 ml	
NA_2 EDTA	4.16 g		
$FeCl_3.6H_2O$	3.15 g		
$CuSO_4.5H_2O$	0.01 g		
$ZnSO_4.7H_2O$	0.022 g		
$CoCl_2.6H_2O$	0.01 g		
$MnCl_2.4H_2O$	0.18 g		
$Na_2MoO_4.2H_2O$	0.006 g		
Vitamin mix		1 ml	
Cyanocobalamin (Vitamin B_{12})	0.0005 g		
Thiamine HCl (Vitamin B_1)	0.1 g		
Biotin	0.0005 g		
Filtered seawater to		1.0 L	

1. Guillard, R. R. L. Culture of phytoplankton for feeding marine invertebrates. In *Culture of marine invertebrate animals*; Springer, 1975; pp. 29–60.
